# Endoscopic ultrasonography-guided hepaticogastrostomy using spatial reality display navigation capable of naked-eye stereopsis

**DOI:** 10.1055/a-2686-3101

**Published:** 2025-09-09

**Authors:** Hirotsugu Maruyama, Yuki Ishikawa-Kakiya, Yuji Kawata, Tatsuya Kurokawa, Yoshinori Shimamoto, Kojiro Tanoue, Yasuhiro Fujiwara

**Affiliations:** 1 12935 Department of Gastroenterology, Graduate School of Medicine, Osaka Metropolitan University, Osaka, Japan

Endoscopic ultrasound-guided hepaticogastrostomy (EUS-HGS) is a growing demand for simpler techniques with higher success rates. Successful insertion of the guidewire (GW) into the bile duct is crucial and should be straightforward. We previously reported a preloaded GW technique using a thin needle 1 . This technique eases GW insertion into the bile duct, but advancing it without cholangiography remains a major challenge.

We addressed this issue by utilizing spatial reality display (SRD) navigation capable of naked-eye stereopsis (Sony, ELF-SR1, Tokyo, Japan). This navigation requires no head-mounted displays such as “mixed reality” technologies 2 3 , enabling multiple users to view 3D images simultaneously and hygienically. Here, we report the case of EUS-HGS using SRD navigation capable the Naked-eye stereopsis.

An 81-year-old man was referred to our hospital for the treatment of obstructive jaundice caused by pancreatic head cancer. Endoscopic retrograde cholangiopancreatography was deemed difficult due to duodenal invasion ( Fig. 1 ); therefore, EUS-HGS was attempted.

**Fig. 1:**
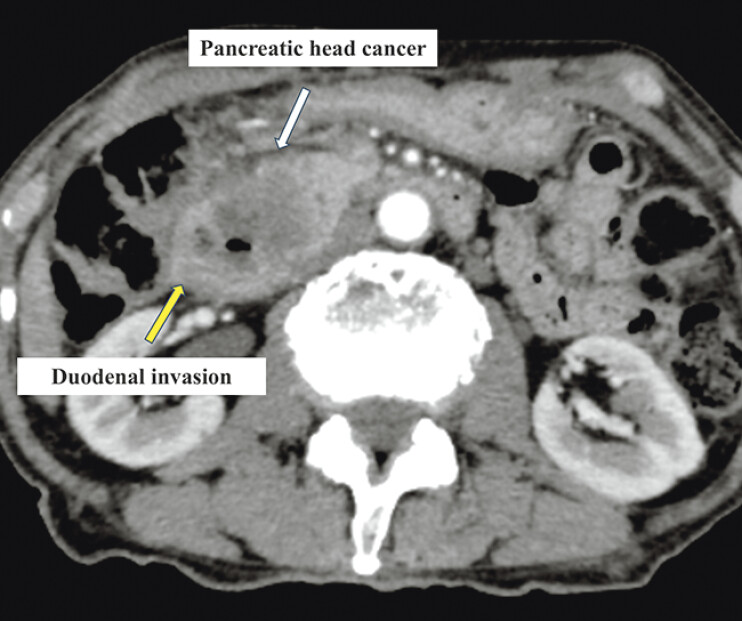
A computed tomography image demonstrating duodenal invasion caused by pancreatic head cancer.

First, the 3D model of the bile duct, the expected GW line when puncturing B2 and B3 and the tip of the EUS was constructed by computed tomography and magnetic resonance cholangiopancreatography using SYNAPSE VINCENT (Fuji Film Medical Co., Ltd., Tokyo, Japan) and were exported as STL files ( Fig. 2 ).

**Fig. 2:**
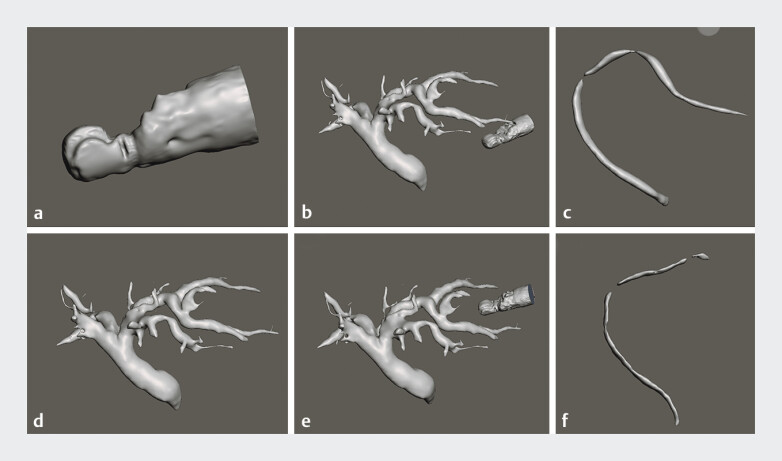
The 3D model was created based on STL (stereolithography) data. **a** Convex-array ultrasound endoscope. **b** Bile duct. **c** Image simulating B3 puncture. **d** Image simulating B2 puncture. **e** Estimated guidewire trajectory after B3 puncture. **f** Estimated guidewire trajectory after B2 puncture.

The model could be easily moved and rotated using a game controller onto SRD, allowing precise alignment of EUS images with actual anatomy ( Fig. 3 ).

**Fig. 3:**
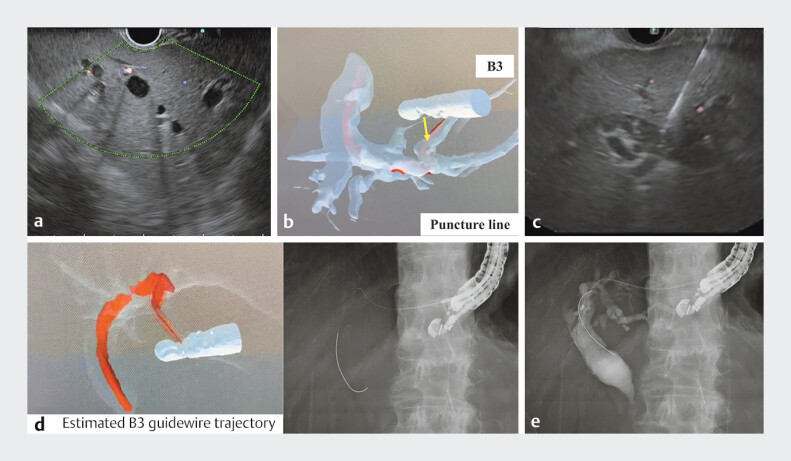
Endoscopic ultrasound-guided hepaticogastrostomy (EUS-HGS) using spatial reality display (SRD) navigation capable of naked-eye stereopsis. **a** EUS image shows dilated intrahepatic bile ducts (B3). **b** By projecting the EUS (convex-array ultrasound endoscope) and bile duct onto the SDR, it is possible to predict the puncture line and angle of B3. (The yellow arrow indicates the puncture line.) **c** EUS image of B3 puncture site. **d** After the EUS (convex-array ultrasound endoscope), the bile duct, and the estimated B3 guidewire trajectory were projected onto the SDR, and the bile duct was made transparent and rotated to match the position in the fluoroscopic image. The actual fluoroscopic image is shown on the right. **e** Fluoroscopic images from the cholangiography are shown.

The EUS-HGS procedure was successfully completed with real-time reference to the SRD navigation ( Video 1 ). No adverse events were observed.

EUS-HGS using SRD navigation capable of naked-eye stereopsis.Video 1

SRD navigation can be used for deep GW insertion during EUS-HGS and for preoperative prediction of the puncture line.

It can be seamlessly integrated into routine endoscopic procedures, allowing visualization of anatomical structures from multiple angles without increasing the physical or cognitive load on the endoscopist.

Endoscopy_UCTN_Code_TTT_1AS_2AH

## Notes

Endoscopy E-Videos https://eref.thieme.de/e-videos
*E-Videos* is an open access online section of the journal *Endoscopy* , reporting on interesting cases and new techniques in gastroenterological endoscopy. All papers include a high-quality video and are published with a Creative Commons CC-BY license. Endoscopy E-Videos qualify for HINARI discounts and waivers and eligibility is automatically checked during the submission process. We grant 100% waivers to articles whose corresponding authors are based in Group A countries and 50% waivers to those who are based in Group B countries as classified by Research4Life (see: https://www.research4life.org/access/eligibility/ ). This section has its own submission website at https://mc.manuscriptcentral.com/e-videos .
